# Comparison of effects of cyclosporine 0.05% and 0.1% in dry eye with Sjögren’s syndrome

**DOI:** 10.1186/s12886-025-04173-x

**Published:** 2025-07-01

**Authors:** Donghyun Jee, Su Yeon Han, Hyun Seung Kim, Eun Chul Kim

**Affiliations:** 1 Department of Ophthalmology, St. Vincent’s Hospital, College of Medicine, The Catholic University of Korea, Seoul, Korea; 2 Department of Ophthalmology, Bucheon St. Mary’s Hospital, College of Medicine, The Catholic University of Korea, Seoul, Korea; 3 Department of Ophthalmology, Seoul St. Mary’s Hospital, College of Medicine, The Catholic University of Korea, Seoul, Korea; 4 Department of Ophthalmology, Bucheon St. Mary’s Hospital, 327 Sosa-ro, Wonmi-gu, Bucheon, Gyeonggi-do 14647 Korea

**Keywords:** Cyclosporine 0.05%, Cyclosporine 0.1%, Sjögren's syndrome

## Abstract

**Purpose:**

To compare the effects of cyclosporine 0.05% and 0.1% in dry eye with Sjögren’s syndrome.

**Methods:**

120 eyes of 60 patients who have been diagnosed with Sjögren’s syndrome were retrospectively enrolled. Thirty patients (group 1, 60 eyes) were treated with cyclosporine 0.1% and 30 patients (group 2, 60 eyes) with cyclosporine 0.05%. Ocular Surface Disease Index Questionnaire (OSDI), Schirmer I test, noninvasive tear break up time (NItBUT), corneal staining score, MMP 9, meibography, meibum quality and expressibility scores, tear meniscus height, and impression cytology were examined before treatment and at 1 and 3 months after treatment.

**Results:**

All of dry eye signs and symptoms of both groups at 1 and 3 months were significantly improved compared with those before treatment, respectively (*P* < 0.05). OSDI, Schirmer I test, NItBUT, corneal and conunctival fluorescein score, MMP-9 grade, goblet cell density, and impression cytology grade of group1 were significantly improved compared with group 2 at 1 and 3 months after treatment (*P* < 0.05). The percentage of discontinued treatment in groups 1 and 2 was 26.7 and 3.3%, respectively.

**Conclusion:**

Cyclosporine 0.1% was more effective for relieving inflammatory dry eye signs and symptoms but less tolerable compared with cyclosporine 0.05% in dry eye with Sjögren’s syndrome.

## Introduction

The Dry Eye Workshop II has previously reported a new definition of dry eye disease (DED): DED is a multifactorial disease of the ocular surface characterized by a loss of homeostasis of the tear film and accompanied by ocular symptoms, in which tear film instability and hyperosmolarity, ocular surface inflammation and damage, and neurosensory abnormalities play aetiological roles [1]. DED is divided into two major subcategories: aqueous-deficient DED and evaporative DED, which are different and distinct, but can often occur together in DED patients. Aqueous deficient DED is characterized by decreased tear production due to lacrimal gland dysfunction, and is commonly associated with Sjögren’s syndrome (SS) [2].

Sjögren’s syndrome (SS) is a chronic autoimmune disease characterized by lymphocytic infiltration of the exocrine glands, specifically the lacrimal gland (LG) and salivary gland (SG) [3, 4]. Because inflammation is important in the etiology of DED, several anti-inflammatory eye drops have been produced.

Topical cyclosporine is one of the useful pharmacologic treatments to treat patients in DED with SS. With the treatment of cyclosporine 0.05% in DED, improvements in blurry vision, decreased frequency of artificial tear use, reduced corneal staining, increased tear (Schirmer I test), reduced inflammatory cytokines and increased goblet cell densities, and reduced HLA-DR expression were reported [5, 6]. Cyclosporine 0.05% was reported to be an effective and safe treatment for patients with primary SS-associated DED [7]. The cyclosporine 0.05% nanoemulsion was reported to improve ocular surface staining scores faster than the conventional cyclosporine 0.05% emulsion [8]. In patients with moderate to severe DED who were unresponsive to cyclosporine 0.05%, shifting to cyclosporine 0.1% improved objective symptoms but resulted in decreased short-term treatment acceptability [9]. In patients with SS-associated DED, switching from cyclosporine 0.05% to cyclosporine 0.1% was reported to be effective in improving ocular symptoms and conjunctival staining [10]. But topical cyclosporine was more effective in the treatment of DED with non-SS than DED with SS in other reports [11].

However, to the best of our knowledge, there was no report about the comparison of the efficacy of cyclosporine 0.05% and 0.1% in Sjögren’s syndrome.

Thus, the objective of this study was to compare the clinical outcomes and efficacy of cyclosporine 0.05% and cyclosporine 0.1% in dry eye with Sjögren’s syndrome.

## Methods

We performed a retrospective data analysis in this study. This study was conducted in compliance with Institutional Review Board regulations and the Declaration of Helsinki. The Institutional Review Board (IRB)/Ethics Committee of Bucheon St. Mary Hospital approved this study protocol (HC23RASI0114). The written informed consent for participation was waived by the IRB committee due to the retrospective nature of the study and the use of anonymized data.

### Inclusion and exclusion criteria

120 eyes of 60 patients who have been diagnosed with Sjögren’s syndrome were enrolled at Bucheon St. Mary Hospital. All patients had symptoms of dry mouth and dry eyes without extraglandular signs. A senior rheumatologist made the diagnosis of Sjögren’s Syndrome in accordance with the 2016 ACR-EULAR Classification Criteria for primary Sjögren’s Syndrome. The final classification criteria are based on the weighted sum of 5 items: anti-SSA/Ro antibody positivity and focal lymphocytic sialadenitis with a focus score of ≥ 1 foci/4 mm2, each scoring 3; an abnormal ocular staining score of ≥ 5 (or van Bijsterveld score of ≥ 4), a Schirmer’s test result of ≤ 5 mm/5 minutes, and an unstimulated salivary flow rate of ≤ 0.1 ml/minute, each scoring 1. Individuals with signs and/or symptoms suggestive of SS who have a total score of ≥ 4 for the above items meet the criteria for primary SS [12]. Patients with secondary SS and subjects using disease modifying medications were excluded from this study. Patients with the following conditions that could affect clinical DE parameters were excluded: those taking medicine for systemic disease such as diabetes mellitus; those with hypertension, allergic disease, thyroid disease, depressive disorder, allergic conjunctivitis, or lid abnormalities; those having undergone ocular surgery within six months before the study; those actively using contact lenses or punctual plugs; and those using topical treatments other than artificial tears within three months before the study or those with any history of topical cyclosporine use.

### Patients’ examination

The patients were divided into two groups; group 1 (30 patients, 60 eyes) was treated with cyclosporine 0.1% (Ikervis^®^; Santen Inc., Osaka, Japan) and group 2 (30 patients, 60 eyes) was treated with cyclosporine 0.05% (Restasis^®^; Allergan Inc., Irvine, CA, USA). Ocular Surface Disease Index Questionnaire (OSDI), noninvasive tear break up time (NItBUT), Schirmer I test, corneal & conjunctival staining scores, tear meniscus height, InflammaDry^®^ test, meibography, meibum quality and expressibility scores, and lipid layer thickness were examined before treatment and at 1 and 3 months after treatment. Corneal & conjunctival staining scores was measured with Oxford scale [13]. The InflammaDry^®^ test was used to grade the expression of matrix metalloproteinase-9 (MMP-9) [14].

### Meibomian gland examination

**Fig. 1:**
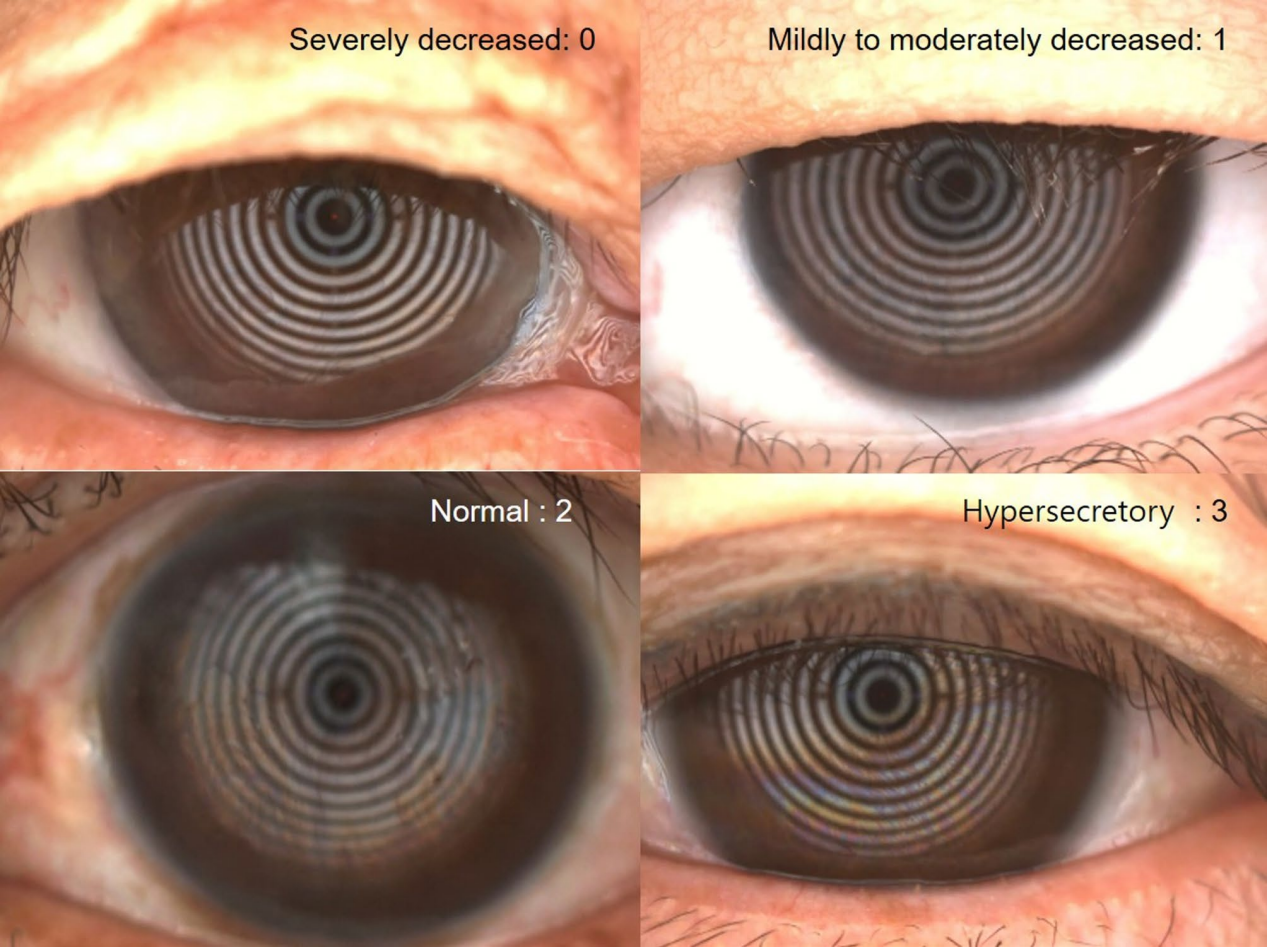
Lipid layer thickness was captured using the Keratograph 5M. Lipid layer thickness was graded from 0 to 3, as follows: 0, severely decreased lipid layer; 1, mildly to moderately decreased lipid layer; 2, normal lipid layer; and 3, hypersecretary lipid layer

Meibomian gland dropout was examined using Keratograph 5 M (Oculus GmbH, Wetzlar, Germany) in the upper and lower lid meibomian glands. The grade of meibomian gland was from 0 to 3 as earlier reported [15]. Meibum expression score (MES) and meibum quality score (MQS) were also graded from 0 to 3 as earlier reported [16]. Lipid layer thickness was examined by Keratograph 5 M, which was graded from 0 to 3, as follows: 0, severely decreased lipid layer; 1, mildly to moderately decreased lipid layer; 2, normal lipid layer; and 3, hypersecretary lipid layer (Fig. 1).

### Impression cytology

The impression cytology was performed on the lower bulbar conjunctiva as earlier described [17]. The goblet cell density was estimated as the number of cells per square millimeter and the degree of squamous metaplasia was graded from 0 to 6 using Tseng’s grading scheme [18].

### Statistical analysis

All statistical analyses were performed using SPSS (version 21.0.1; SPSS Inc., Chicago, IL). A sample size calculation was based on research published regarding the incidence of Sjögren’s syndrome in Korea [19]. A power analysis was performed for each primary outcome to strengthen the study design by using G Power 3.1.9.4 software [20]. Generalized estimating equation (GEE) was used to adjust the correlation between right and left eyes [21]. Comparisons between two groups were performed with Mann–Whitney tests. The Wilcoxon signed rank test was used to compare the data before and after treatment. *P* values < 0.05 were considered statistically significant.

## Results

There were no statistically significant differences between the two groups according to age, duration of disease, OSDI, tear height, the expression of matrix metalloproteinase-9 (MMP-9), Schirmer I test, NItBUT, corneal and conjunctival staining score, meibomian gland score, lipid layer thickness grade, goblet cell density, or impression cytological grade (*p* > 0.05) (Table 1).

**Table 1:** Data of patients before treatment

Parameter	Cyclosporine 0.1% (Group 1)	Cyclosporine 0.05% (Group 2)
Number of eyes	**60**	**60**
F: M	30:0	30:0
Age, years (range)	50.4 ± 10.6 (25–69)	53.58 ± 13.80 (27–83)
Duration of disease, years (range)	2.17 ± 0.53 (0–9)	1.3 ± 0.46 (0.1–7.7)
OSDI score (range)	34.82 ± 9.87 (25–43)	36.54 ± 10.25 (26–46)
Tear Height, mm (range)	0.18 ± 0.07 (0.09–0.49)	0.21 ± 0.10 (0.08–0.51)
MMP-9 grade (range)	1.17 ± 1.43 (0–4)	1.46 ± 1.44 (0–4)
Schirmer I, mm (range)	2.17 ± 3.61 (0–15)	2.83 ± 4.25 (0–15)
NItBUT, sec (range)	5.78 ± 3.16 (2.68–15.1)	5.66 ± 4.45 (1.91–23.9)
Fluorescein Stain (range)	3.13 ± 1.42 (0–5)	3.04 ± 1.54 (0–5)
Meiboscore (range)	0.80 ± 0.86 (0–3)	0.62 ± 0.77 (0–3)
Meibum expressibility (range)	1.25 ± 0.78 (0–3)	1.21 ± 0.90 (0–3)
Meibum quality (range)	1.34 ± 0.52 (0–3)	1.38 ± 0.61 (0–3)
Lipid layer thickness (range)	1.54 ± 0.72 (0–3)	1.50 ± 0.72 (0–3)
Goblet cell density (cell/mm^2^) (range)	98.37 ± 40.58 (57–138)	103.25 ± 42.65 (61–145)
Impression cytological grade (range)	3.47 ± 1.25 (2–6)	3.51 ± 1.35 (2–6)

There was no statistical difference between the two groups (*p* > 0.05)

OSDI: Ocular Surface Disease Index Questionnaire

MMP-9: Matrix metalloproteinase-9

NItBUT: Noninvasive tear break up time

Fluorescein Stain: corneal & conjunctival fluorescein staining score

Values are presented as mean ± SD. D; diopter

### OSDI score

**Fig. 2:**
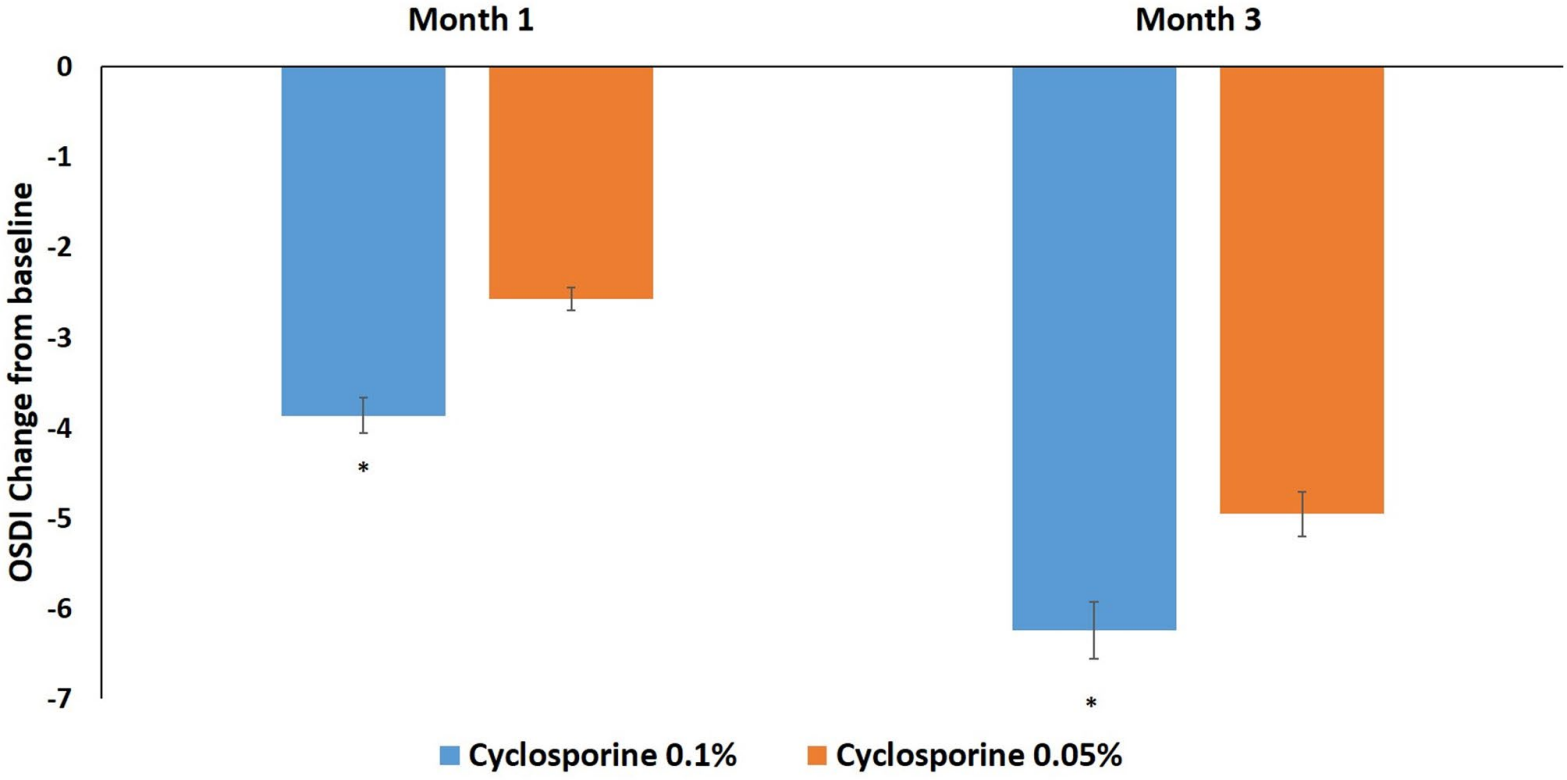
OSDI score change of both groups after treatment. Mean value ± standard error. Ocular Surface Disease Index Questionnaire (OSDI) score change from baseline in group 1 was significantly decreased compared with those in groups 2 at 1 and 3 months after treatment, respectively (*P* < 0.05)

OSDI scores of both groups at 1 and 3 months after treatment were significantly decreased compared with those before treatment, respectively (*P* < 0.05). OSDI score change from baseline in group 1 (-3.86 ± 0.98 and − 6.24 ± 1.24) was significantly decreased compared with that in group 2 (-2.57 ± 0.85 and − 4.95 ± 1.16) at 1 and 3 months after treatment, respectively (*P* < 0.05) (Fig. 2).

### Schirmer i test and NItBUT

**Fig. 3:**
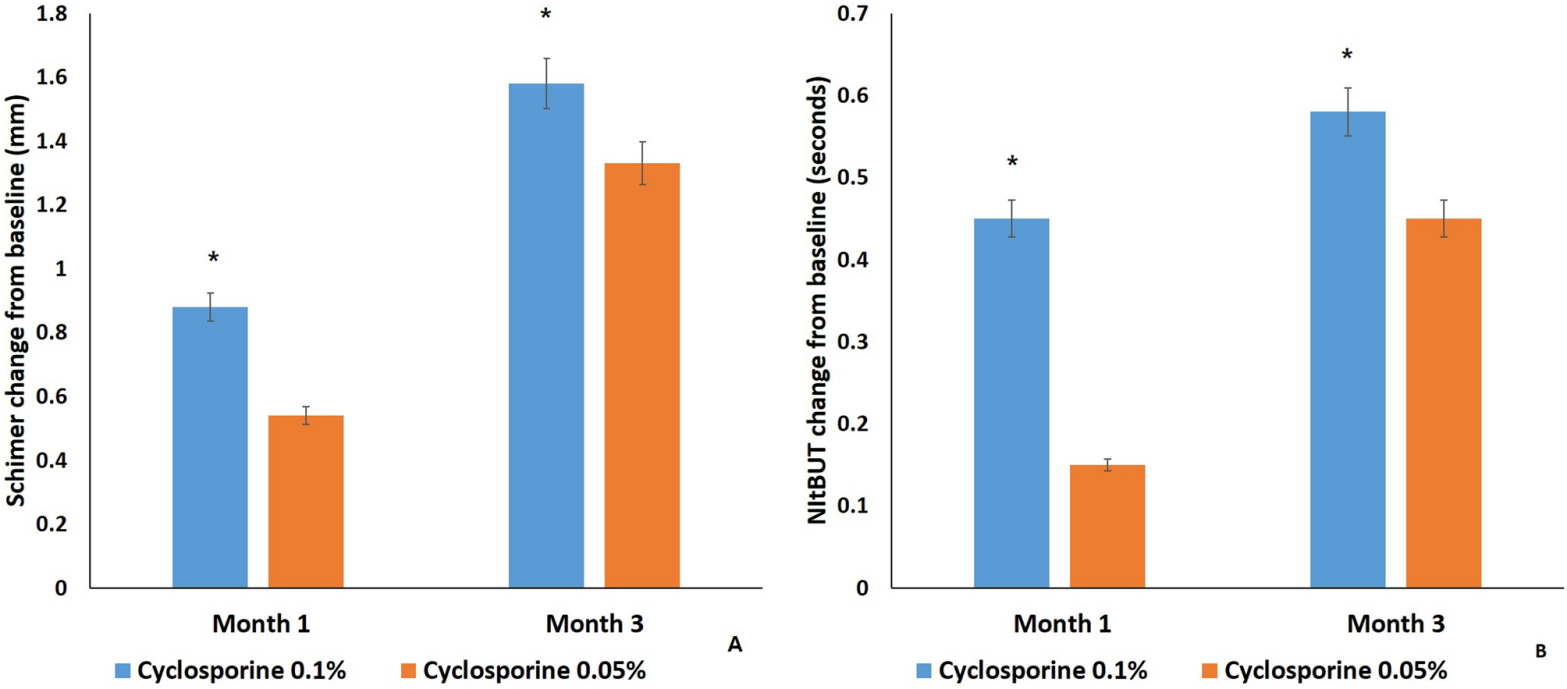
Schirmer I and NItBUT score changes of both groups after treatment. Mean value ± standard error. Schirmer I (**A**) and noninvasive tear break up time (NItBUT) (**B**) change from baseline in group 1 were significantly increased compared with those in group 2 at 1 and 3 months after treatment, respectively (*P* < 0.05)

Schirmer I (mm) and NItBUT (seconds) of both groups at 1 and 3 months after treatment were significantly increased compared with those before treatment, respectively (*P* < 0.05). Schirmer I change from baseline in group 1 (0.88 ± 0.16 and 1.58 ± 0.16) was significantly increased compared with that in group 2 (0.54 ± 0.15 and 1.33 ± 0.17) at 1 and 3 months after treatment, respectively (*P* < 0.05) (Fig. 3A). NItBUT change from baseline (seconds) of group 1 (0.45 ± 0.11 and 0.58 ± 0.12) was significantly increased compared with that of group 2 (0.15 ± 0.17 and 0.45 ± 0.18) at 1 and 3 months after treatment, respectively (*P* < 0.05) (Fig. 3B).

### Corneal and conjunctival staining and MMP-9

**Fig. 4:**
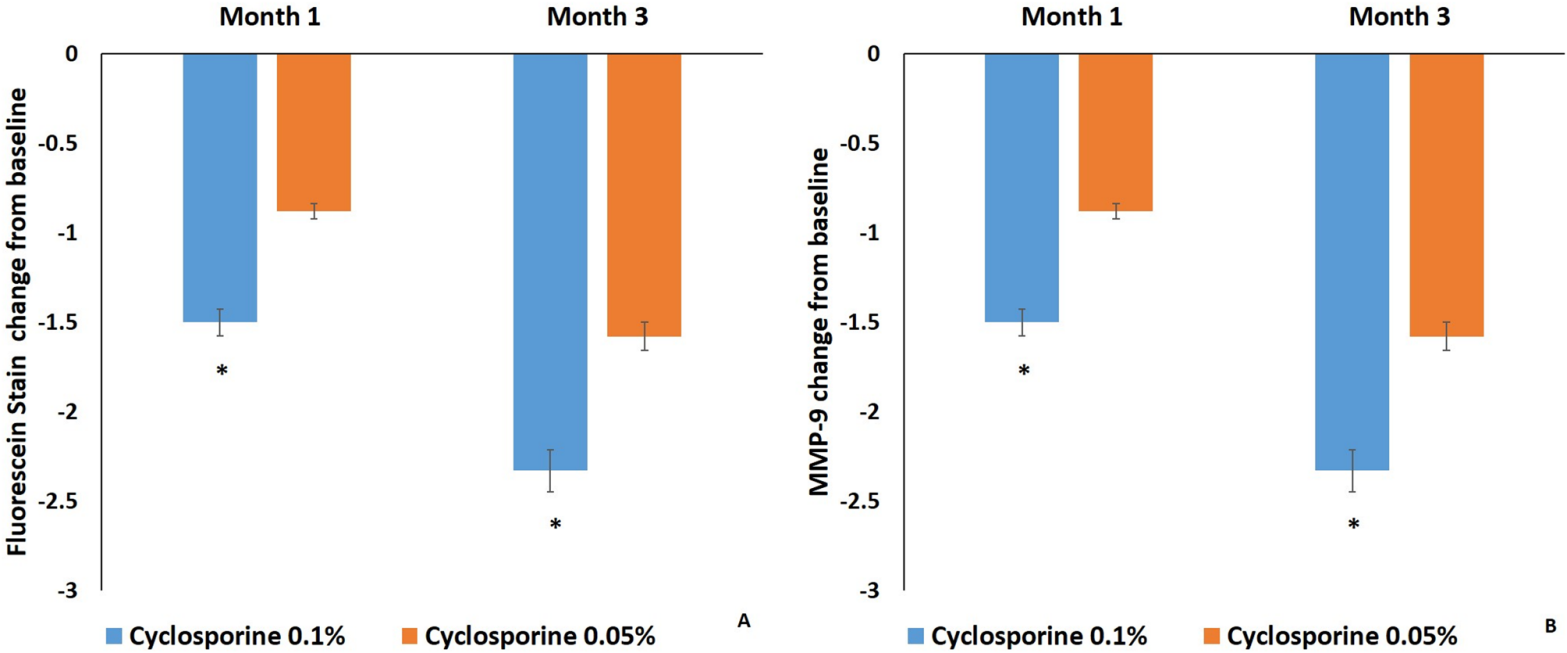
Fluorescein score and MMP-9 grade change of both groups after treatment. Mean value ± standard error. Corneal and conjunctival fluorescein score (**A**) and MMP-9 grade (**B**) change from baseline in group 1 were significantly decreased compared with those in group 2 at 1 and 3 months after treatment, respectively (*P* < 0.05)

Corneal and conjunctival fluorescein scores and MMP-9 grades of both groups at 1 and 3 months after treatment were significantly decreased compared with those before treatment, respectively (*P* < 0.05). Corneal and conjunctival fluorescein score change from baseline in group 1 (-1.50 ± 0.18, -2.33 ± 0.26) was significantly decreased compared with that in group 2 (-0.88 ± 0.16 and − 1.58 ± 0.26) at 1 and 3 months after treatment, respectively (*P* < 0.05) (Fig. 4A). MMP-9 grade change from baseline (seconds) in group 1 (-0.92 ± 0.16 and − 1.46 ± 0.21) was significantly decreased compared with that in group 2 (-0.42 ± 0.15 and − 0.88 ± 0.16) at 1 and 3 months after treatment, respectively (*P* < 0.05) (Fig. 4B).

### Meibomian gland score

**Fig. 5:**
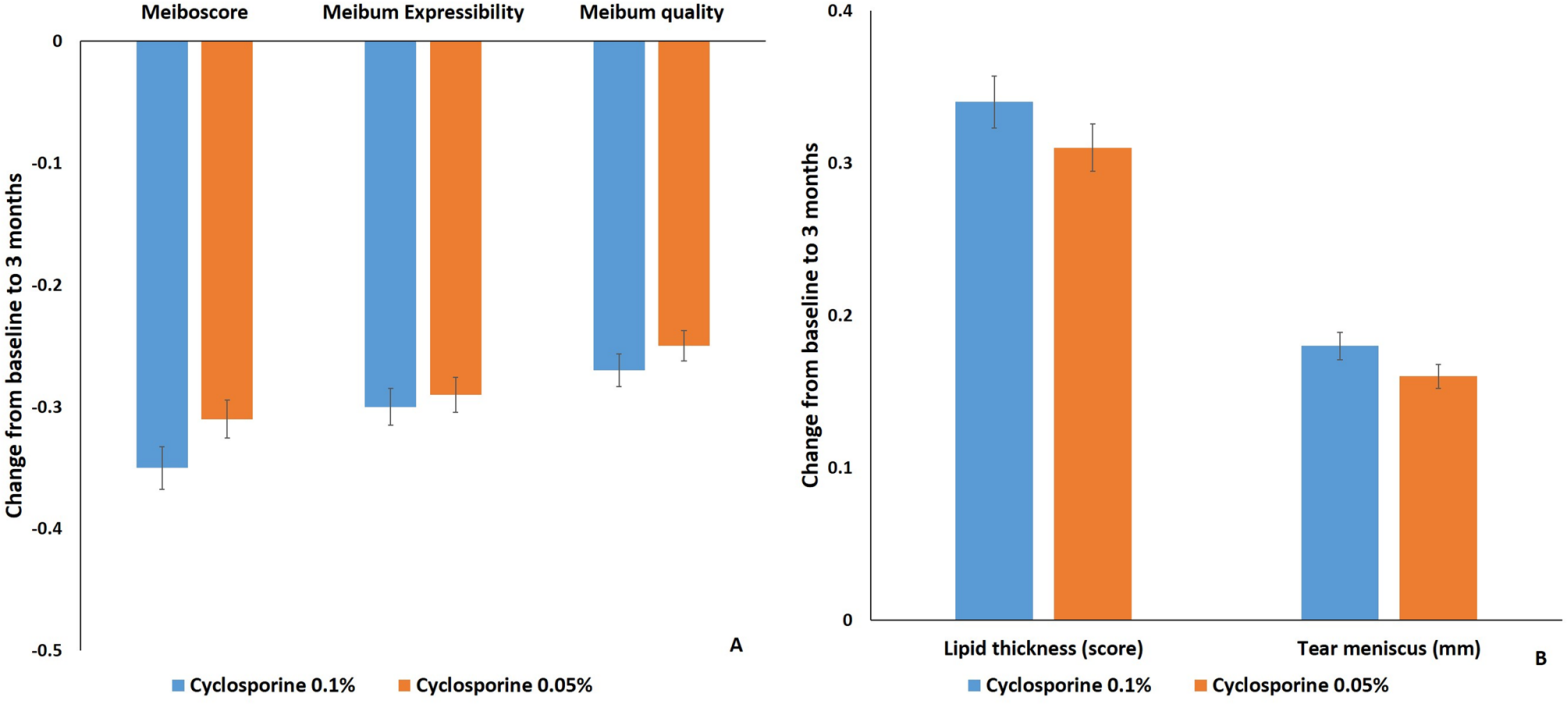
Meibomian gland, lipid thickness, and tear height change after treatment. Mean value ± standard error. There was no significant difference in meiboscore, meibum expressibility, meibum quality (**A**), lipid thickness grade, or tear meniscus height (**B**) between both groups at 3 months after treatment (*P* > 0.05)

Meiboscore, meibum expressibility, and meibum quality in both groups at 3 months after treatment were significantly improved compared with those before treatment, respectively (*P* < 0.05). But there was no significant difference in meiboscore, meibum expressibility, or meibum quality between both groups at 3 months after treatment (*P* > 0.05) (Fig. 5A).

### Lipid thickness and tear meniscus height

Lipid thickness grade and tear meniscus height (mm) of both groups at 1 and 3 months after treatment were significantly improved compared with those before treatment, respectively (*P* < 0.05). But there was no significant difference in lipid thickness grade or tear meniscus height between both groups at 3 months after treatment (*P* > 0.05) (Fig. 5B).

### Impression cytology

**Fig. 6:**
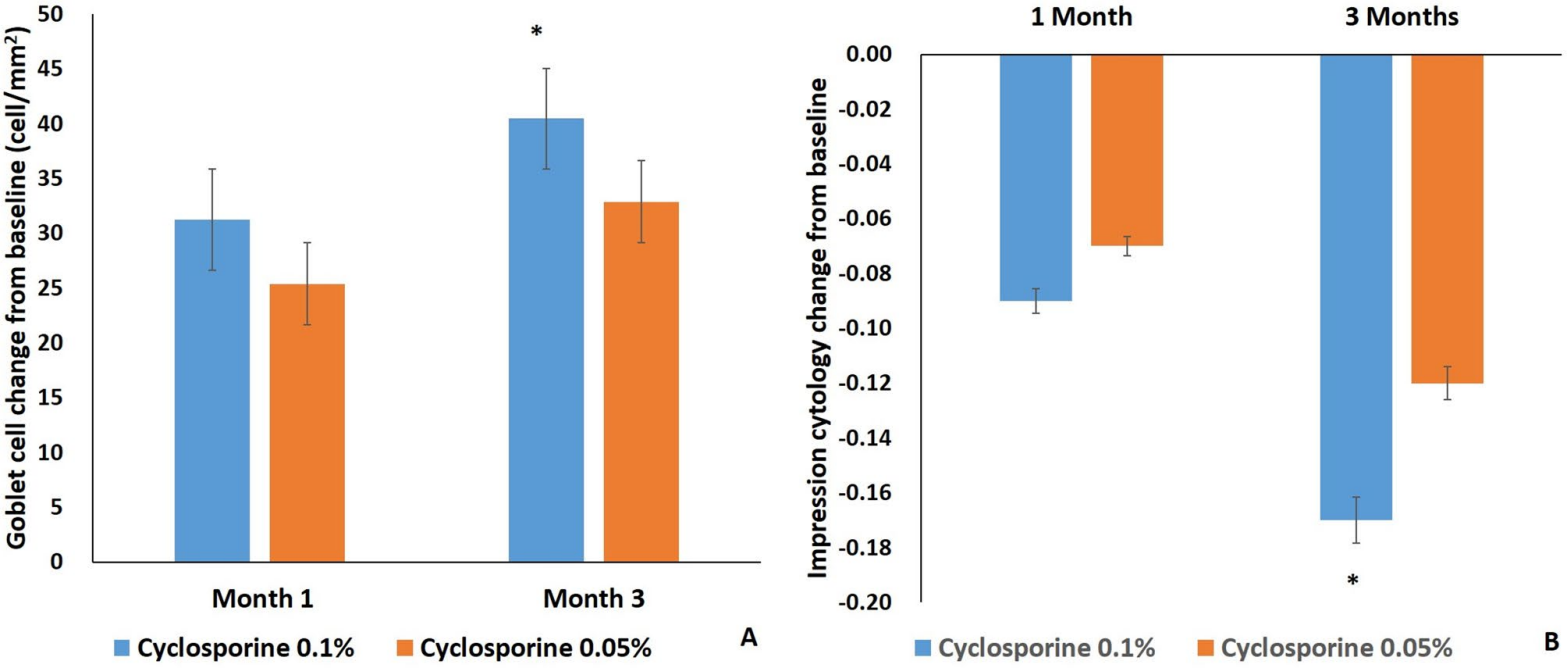
Goblet cell density (**A**) and impression cytology grade (**B**) change from baseline. Mean value ± standard error. The change of goblet cell density in group 1 was significantly increased and the change of impression cytology grade in groups 1 was significantly decreased compared with group 2 at 3 months after treatment (*P* < 0.05)

The change of goblet cell density in group 1 (70.36 ± 10.25) was significantly increased compared with group 2 (50.65 ± 8.27) at 3 months after treatment (*P* < 0.05) (Fig. 6A). The change of impression cytology grade in groups 1 (-0.17 ± 0.08) was significantly decreased compared with group 2 (-0.12 ± 0.07) at 3 months after treatment (*P* < 0.05) (Fig. 6B).

**Fig. 7:**
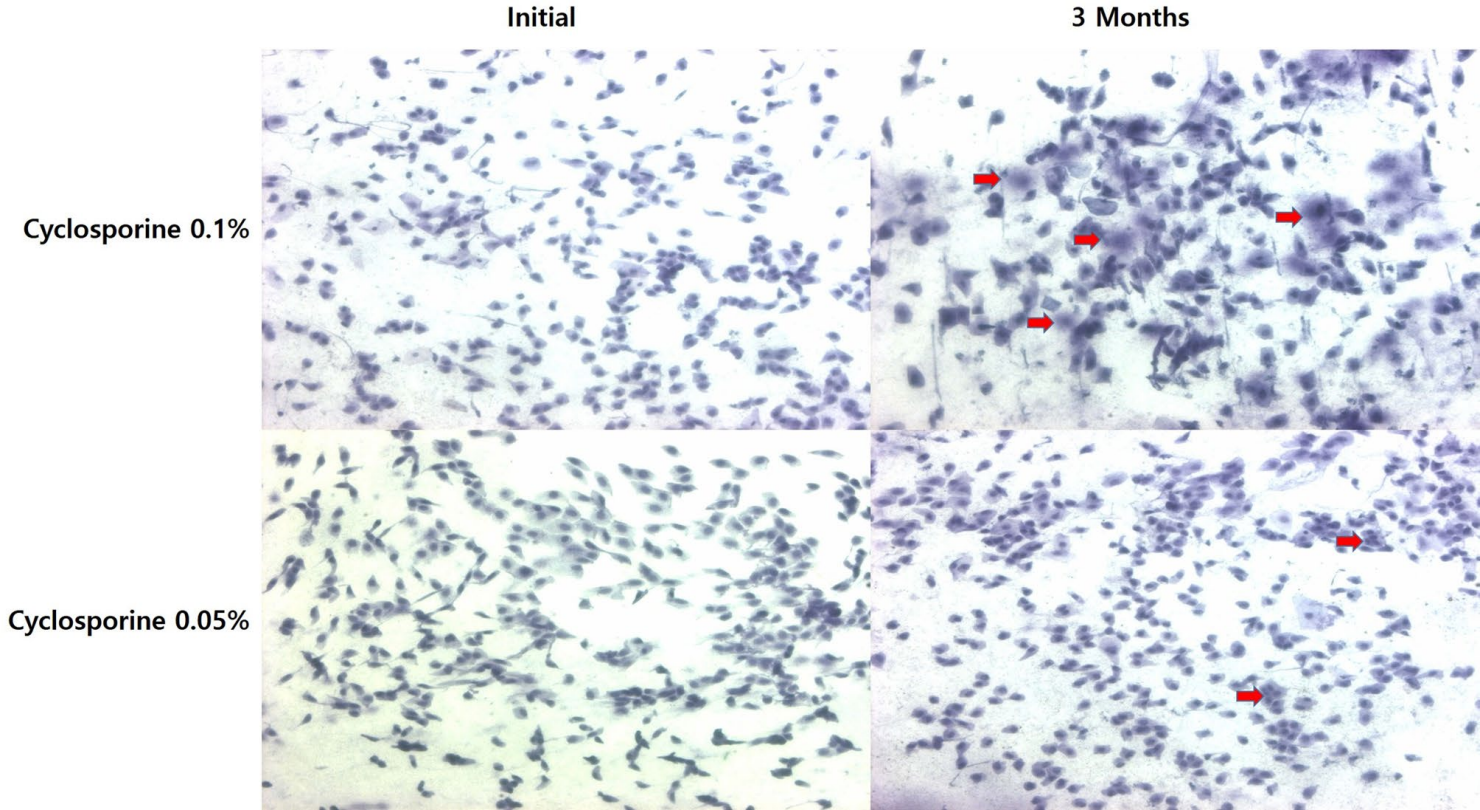
Clinical pictures of impression cytology in each group at the initial evaluation and at 3 months. At 3 months, there was an increase in goblet cells (red arrows) from the initial evaluation in group 1 versus group 2 (×100, PAS-hematoxylin staining)

At 3 months, there was an increase in goblet cells (red arrows) from the initial evaluation in group 1 versus group 2 (×100, PAS-hematoxylin staining) (Fig. 7).

### Discontinue treatment

The percentage of discontinued treatment in group 1 was 26.7% (8 patients). The reasons for the discontinued treatment in group 1 were ocular pain (*n* = 5), conjunctival hyperemia (*n* = 2), and tearing (*n* = 1). The percentage of the discontinued treatment in group 2 was 3.3% (1 patient). The reason for the discontinued treatment in group 2 was ocular pain.

## Discussion

Sjögren’s syndrome (SS) is an autoimmune disease that affect the exocrine glands, particularly the lacrimal glands [22, 23]. SS can cause chronic severe dry eye (DE) because of the lymphocytic infiltration of in the lacrimal glands and conjunctiva [24–26]. Treatment of DED is challenging due to a vicious cycle of tear film instability, hyperosmolarity, and ocular surface inflammation [27–29].

Because inflammation is so important in the development of DED, many anti-inflammatory medications have been produced [2]. Cyclosporine A (CsA), a calcineurin inhibitor, is used to treat inflammatory diseases of the ocular surface, by inhibiting the T lymphocytes and inflammatory cytokines [5, 30, 31]. Cyclosporine A has also shown significant improvement of signs and symptoms in moderate-to-severe DED [32].

Cyclosporine 0.05% was reported to be an effective and safe treatment for primary SS with dry eye [7]. However, Cubuk M.O. et al. reported that cyclosporine was more effective in the treatment of dry eye patients with non-SS than those with SS [11]. But topical CsA was reported to increase corneal sub-basal nerve density, improving clinical signs and symptoms of dry eye associated with SS [33]. Supra-lacrimal protein-based carriers for CsA were reported to decrease Th17-mediated autoimmunity in a murine model of SS [34].

When applied topically as an oil-based formulation (anionic emulsion), CsA has low bioavailability and is poorly tolerated [35]. Topical CsA 0.05% in nanoemulsion form enhances the bioavailability of drugs with poor aqueous solubility [8]. Both nanoemulsion and oil-based cyclosporine A improved ocular signs, symptoms, and conjunctival inflammation. However, the cyclosporine A nanoemulsion improved ocular surface staining scores faster than the oil-based emulsion [8]. CsA 0.1% cationic emulsion (CE) was designed to increase the retention time of CsA on the cornea and conjunctiva by interacting with cationic surfactants and the negatively charged mucin in the tear film [36]. CsA CE significantly improved signs and symptoms in patients with moderate-to-severe DED and patients with SS with severe DED [32]. But In patients with moderate to severe DED who were unresponsive to 0.05% cyclosporine, shifting to 0.1% cyclosporine was reported to improve objective signs but with lower treatment tolerability in the short term [9]. In our result, the percentage of the discontinued treatment in groups 1 and 2 was 26.7% and 3.3%, respectively (Table 2). In patients with SS-associated DE, switching from CsA anionic emulsion (AE) to CsA cationic emulsion (CE) improved ocular symptoms and conjunctival staining. Furthermore, corneal staining reduced in patients with severe keratitis [10]. In our study, Schirmer I (mm), NItBUT (seconds), corneal & conjunctival staining, MMP-9, meibomian gland score, lipid thickness, and tear meniscus height of both CsA 0.05% and 0.1% groups at 1 and 3 months after treatment were significantly improved compared with those before treatment, respectively (*P* < 0.05). MMP-9 is an inflammatory molecule that has a role in the mechanisms of dry eye associated with Sjögren’s syndrome [23]. MMP-9 promotes corneal epithelial healing process by modulating the inflammatory response [37]. MMP-9 expression and grade were reported to be lower after cyclosporine treatment than after diquafosol treatment in dry eye disease [14]. In this study, MMP-9 grade was significantly improved after treatment with CsA 0.05% and 0.1%.

**Table 2:** Percentage of discontinued treatment in both groups

Parameter	0.1% Cyclosporine (Group 1)	0.05% Cyclosporine (Group 2)
Number of patients	**30**	**30**
Discontinued treatment (N)	8	1
Percentage of discontinued treatment	26.7%	3.3%

The percentage of discontinued treatment in groups 1 and 2 was 26.7% (8 patients) and 3.3% (1 patient), respectively

Several studies reported the efficacy of CsA 0.05% or CsA 0.1% in dry eye with Sjögren’s syndrome. But, to the best of our knowledge, there was no study about the comparison of the efficacy of cyclosporine 0.05% and 0.1% in dry eye with Sjögren’s syndrome.

In this study, all dry eye signs and symptoms in both groups at 1 and 3 months were significantly improved compared with those before treatment, respectively (*P* < 0.05). OSDI, Schirmer I test, NItBUT, corneal and conjunctival fluorescein score, and MMP-9 grade of group 1 were significantly improved compared with those of group 2 at 1 and 3 months after treatment (*P* < 0.05) (Fig. 2-4). But there was no significant difference in meiboscore, meibum expressibility, meibum quality, lipid thickness, or tear meniscus height between both groups at 3 months after treatment (*P* > 0.05) (Fig. 5).

Meibum quality score was improved with treatment of cyclosporine 0.1% compared with control group in dry eye syndrome with meibomian gland dysfunction [38]. And lid margin telangiectasia was improved better in the cyclosporine 0.05% group than in the control group at 3months in dry eye syndrome with meibomian gland dysfunction [39]. With our results, we believe that both cyclosporine 0.05% and 0.1% can relieve Meibomian gland inflammation and that their therapeutic effects are similar in Sjögren’s syndrome.

The change in goblet cell density in group 1 significantly increased, and the change in impression cytology grade in group 1 significantly decreased compared with group 2 at 3 months after treatment (*P* < 0.05) (Figs. 6 and 7).

Many previous studies have demonstrated the effectiveness of cyclosporine in dry eye patients with Sjögren’s syndrome. Switching study from cyclosporine A 0.05% to cyclosporine A 0.1% in patients with dry Eye associated with Sjögren’s syndrome was reported [10]. But there was no report about the comparison of the efficacy of cyclosporine 0.05% and 0.1% in Sjögren’s syndrome. In this study, cyclosporine 0.1% was more effective in improving anti-inflammatory symptoms and signs than cyclosporine 0.05% in dry eye with Sjögren’s syndrome.

This retrospective, non-blinded, chart review-based study may be limited by group selection bias and assessment bias due to its design. A prospective approach was not feasible initially due to the need for patient consent and significant funding, though future studies should prioritize randomized, double-blind placebo-controlled prospective designs to strengthen validity. Additionally, all participants were female, reflecting Sjögren’s syndrome’s higher prevalence in women, but this introduces potential gender-related bias in generalizing results. Cyclosporine 0.1% eyedrops may cause ocular pain, and exclusion of patients with severe discomfort could have artificially lowered OSDI scores in Group 1, skewing outcomes. A crossover clinical trial evaluating cyclosporine 0.05% and 0.1% across different treatment periods could provide deeper insights into short- and long-term efficacy, informing tailored therapy strategies for dry eye disease (DED) patients.

In conclusion, both cyclosopine 0.05% and 0.1% improved the signs and symptoms of patients with dry eye with Sjögren’s syndrome. OSDI, Schirmer I test, NItBUT, corneal and conjunctival fluorescein score, MMP-9 grade, goblet cell density, and impression cytology grade of cyclosopine 0.1% were significantly improved compared with cyclosopine 0.05%. Cyclosopine 0.1% was more effective in improving anti-inflammatory symptoms and signs than cyclosopine 0.05% in dry eye with Sjögren’s syndrome. Because of the side effects such as ocular pain, conjunctival hyperemia, and tearing, cyclosopine 0.1% was more intolerant than cyclosopine 0.05%, even in dry eye with Sjögren’s syndrome.

## Data Availability

The datasets used and/or analyzed during the current study available from the corresponding author on reasonable request.

## References

[1] Craig JP, Nichols KK, Akpek EK, Caffery B, Dua HS, Joo CK, Liu Z, Nelson JD, Nichols JJ, Tsubota K, et al. TFOS DEWS II definition and classification report. Ocul Surf. 2017;15(3):276–83. 28736335 10.1016/j.jtos.2017.05.008

[2] Kuklinski E, Asbell PA. Sjogren’s syndrome from the perspective of ophthalmology. Clin Immunol. 2017;182:55–61. 28476437 10.1016/j.clim.2017.04.017

[3] Dawczynski C, Hackermeier U, Viehweger M, Stange R, Springer M, Jahreis G. Incorporation of n-3 PUFA and γ-linolenic acid in blood lipids and red blood cell lipids together with their influence on disease activity in patients with chronic inflammatory arthritis–a randomized controlled human intervention trial. Lipids Health Dis. 2011;10:130. 21816071 10.1186/1476-511X-10-130 PMC3162909

[4] Nguyen CQ, Peck AB. Unraveling the pathophysiology of Sjogren syndrome-associated dry eye disease. Ocul Surf. 2009;7(1):11–27. 19214349 10.1016/s1542-0124(12)70289-6 PMC2861866

[5] Sall K, Stevenson OD, Mundorf TK, Reis BL. Two multicenter, randomized studies of the efficacy and safety of cyclosporine ophthalmic emulsion in moderate to severe dry eye disease. CsA phase 3 study group. Ophthalmology. 2000;107(4):631–9. 10768324 10.1016/s0161-6420(99)00176-1

[6] Kunert KS, Tisdale AS, Stern ME, Smith JA, Gipson IK. Analysis of topical cyclosporine treatment of patients with dry eye syndrome: effect on conjunctival lymphocytes. Arch Ophthalmol. 2000;118(11):1489–96. 11074805 10.1001/archopht.118.11.1489

[7] Gao M, Zhao L, Liang R, Zhu Q, Zhao Q, Kong X. Evaluation of the efficacy and safety of topical 0.05% cyclosporine eye drops (II) in the treatment of dry eye associated with primary sjögren’s syndrome. Ocul Immunol Inflamm. 2023;31(8):1662–8. 35914303 10.1080/09273948.2022.2094812

[8] Kang MJ, Kim YH, Chou M, Hwang J, Cheon EJ, Lee HJ, Chung SH. Evaluation of the efficacy and safety of A novel 0.05% cyclosporin A topical nanoemulsion in primary sjögren’s syndrome dry eye. Ocul Immunol Inflamm. 2020;28(3):370–8. 30986119 10.1080/09273948.2019.1587470

[9] Chan YH, Sun CC. Efficacy and safety of topical cyclosporine 0.1% in moderate-to-severe dry eye disease refractory to topical cyclosporine 0.05% regimen. Taiwan J Ophthalmol. 2023;13(1):68–74. 37252163 10.4103/tjo.TJO-D-22-00140 PMC10220438

[10] Kim J, Moon TK, Yoon HJ, Ji YS, Yoon KC. Efficacy of switching from cyclosporine A 0.05% anionic emulsion to cyclosporine A 0.1% cationic emulsion in patients with dry eye associated with sjögren’s syndrome. J Ocul Pharmacol Ther. 2021;37(8):472–8. 34449255 10.1089/jop.2020.0146

[11] Cubuk MO, Ucgul AY, Ozgur A, Ozulken K, Yuksel E. Topical cyclosporine a (0.05%) treatment in dry eye patients: a comparison study of sjogren’s syndrome versus non-Sjogren’s syndrome. Int Ophthalmol. 2021;41(4):1479–85. 33484384 10.1007/s10792-021-01708-1

[12] Shiboski CH, Shiboski SC, Seror R, Criswell LA, Labetoulle M, Lietman TM, Rasmussen A, Scofield H, Vitali C, Bowman SJ, et al. 2016 American college of rheumatology/european league against rheumatism classification criteria for primary sjögren’s syndrome: A consensus and Data-Driven methodology involving three international patient cohorts. Arthritis Rheumatol. 2017;69(1):35–45. 27785888 10.1002/art.39859 PMC5650478

[13] Bron AJ, Evans VE, Smith JA. Grading of corneal and conjunctival staining in the context of other dry eye tests. Cornea. 2003;22(7):640–50. 14508260 10.1097/00003226-200310000-00008

[14] So HR, Baek J, Lee JY, Kim HS, Kim MS, Kim EC. Comparison of matrix metallopeptidase-9 expression following cyclosporine and Diquafosol treatment in dry eye. Ann Med. 2023;55(1):2228192. 37354028 10.1080/07853890.2023.2228192 PMC10291919

[15] Srinivasan S, Menzies K, Sorbara L, Jones L. Infrared imaging of meibomian gland structure using a novel keratograph. Optom Vis Sci. 2012;89(5):788–94. 22525129 10.1097/OPX.0b013e318253de93

[16] Chan HH: Is the peripheral retina an important site for myopic development? [Liu, Wildsoet Y. C (2011) The effectof two-zone concentric bifocal spectacle lenses on refractive error development and eye growth in young chicks. Invest Ophthalmol Vis Sci 52(2):1078–1086]. *Graefes Arch Clin Exp Ophthalmol* 2011, 249(7):955–956. 10.1007/s00417-011-1720-y 21643721

[17] Anshu, Munshi MM, Sathe V, Ganar A. Conjunctival impression cytology in contact lens wearers. Cytopathology. 2001;12(5):314–20. 11722511 10.1046/j.1365-2303.2001.00349.x

[18] Tseng SC. Staging of conjunctival squamous metaplasia by impression cytology. Ophthalmology. 1985;92(6):728–33. 3897935 10.1016/s0161-6420(85)33967-2

[19] Kim HJ, Kim KH, Hann HJ, Han S, Kim Y, Lee SH, Kim DS, Ahn HS. Incidence, mortality, and causes of death in physician-diagnosed primary sjögren’s syndrome in korea: A nationwide, population-based study. Semin Arthritis Rheum. 2017;47(2):222–7. 28729155 10.1016/j.semarthrit.2017.03.004

[20] Faul F, Erdfelder E, Lang AG, Buchner A. G*Power 3: a flexible statistical power analysis program for the social, behavioral, and biomedical sciences. Behav Res Methods. 2007;39(2):175–91. 17695343 10.3758/bf03193146

[21] Liang KY, Zeger SL. Regression analysis for correlated data. Annu Rev Public Health. 1993;14:43–68. 8323597 10.1146/annurev.pu.14.050193.000355

[22] Moutsopoulos HM, Chused TM, Mann DL, Klippel JH, Fauci AS, Frank MM, Lawley TJ, Hamburger MI. Sjögren’s syndrome (Sicca syndrome): current issues. Ann Intern Med. 1980;92(2 Pt 1):212–26. 7352730 10.7326/0003-4819-92-2-212

[23] The definition and classification of dry eye disease: report of the Definition and Classification Subcommittee of the International Dry Eye WorkShop. (2007). *Ocul Surf* 2007, 5(2):75–92. 10.1016/s1542-0124(12)70081-2 17508116

[24] Raphael M, Bellefqih S, Piette JC, Le Hoang P, Debre P, Chomette G. Conjunctival biopsy in sjögren’s syndrome: correlations between histological and immunohistochemical features. Histopathology. 1988;13(2):191–202. 3169687 10.1111/j.1365-2559.1988.tb02024.x

[25] Hikichi T, Yoshida A, Tsubota K. Lymphocytic infiltration of the conjunctiva and the salivary gland in sjögren’s syndrome. Arch Ophthalmol. 1993;111(1):21–2. 8424715 10.1001/archopht.1993.01090010023009

[26] Pflugfelder SC, Huang AJ, Feuer W, Chuchovski PT, Pereira IC, Tseng SC. Conjunctival cytologic features of primary sjögren’s syndrome. Ophthalmology. 1990;97(8):985–91. 1698273 10.1016/s0161-6420(90)32478-8

[27] Baudouin C, Aragona P, Messmer EM, Tomlinson A, Calonge M, Boboridis KG, Akova YA, Geerling G, Labetoulle M, Rolando M. Role of hyperosmolarity in the pathogenesis and management of dry eye disease: proceedings of the OCEAN group meeting. *Ocul Surf* 2013, 11(4):246–258. 10.1016/j.jtos.2013.07.003 24112228

[28] Baudouin C, Irkeç M, Messmer EM, Benítez-Del-Castillo JM, Bonini S, Figueiredo FC, Geerling G, Labetoulle M, Lemp M, Rolando M, et al. Clinical impact of inflammation in dry eye disease: proceedings of the ODISSEY group meeting. Acta Ophthalmol. 2018;96(2):111–9. 28390092 10.1111/aos.13436 PMC5836968

[29] Kim M, Kim HS, Na KS. Correlation between tear osmolarity and other ocular surface parameters in primary sjögren’s syndrome. Korean J Ophthalmol. 2017;31(1):25–31. 28243020 10.3341/kjo.2017.31.1.25 PMC5327171

[30] Brignole F, Pisella PJ, De Saint Jean M, Goldschild M, Goguel A, Baudouin C. Flow cytometric analysis of inflammatory markers in KCS: 6-month treatment with topical cyclosporin A. Invest Ophthalmol Vis Sci. 2001;42(1):90–5. 11133852

[31] Foulks GN. Topical cyclosporine for treatment of ocular surface disease. Int Ophthalmol Clin. 2006;46(4):105–22. 17060797 10.1097/01.iio.0000212135.77675.6a

[32] Leonardi A, Messmer EM, Labetoulle M, Amrane M, Garrigue JS, Ismail D, Sainz-de-la-Maza M, Figueiredo FC, Baudouin C. Efficacy and safety of 0.1% ciclosporin A cationic emulsion in dry eye disease: a pooled analysis of two double-masked, randomised, vehicle-controlled phase III clinical studies. Br J Ophthalmol. 2019;103(1):125–31. 29545413 10.1136/bjophthalmol-2017-311801 PMC6317444

[33] Levy O, Labbé A, Borderie V, Hamiche T, Dupas B, Laroche L, Baudouin C, Bouheraoua N. Increased corneal sub-basal nerve density in patients with Sjögren syndrome treated with topical cyclosporine A. Clin Exp Ophthalmol. 2017;45(5):455–63. 27957797 10.1111/ceo.12898

[34] Guo H, Ju Y, Choi M, Edman MC, Louie SG, Hamm-Alvarez SF, MacKay JA. Supra-lacrimal protein-based carriers for cyclosporine A reduce Th17-mediated autoimmunity in murine model of sjögren’s syndrome. Biomaterials. 2022;283:121441. 35306230 10.1016/j.biomaterials.2022.121441 PMC8982551

[35] Lallemand F, Felt-Baeyens O, Besseghir K, Behar-Cohen F, Gurny R. Cyclosporine A delivery to the eye: a pharmaceutical challenge. Eur J Pharm Biopharm. 2003;56(3):307–18. 14602172 10.1016/s0939-6411(03)00138-3

[36] Boboridis KG, Konstas AGP. Evaluating the novel application of cyclosporine 0.1% in ocular surface disease. Expert Opin Pharmacother. 2018;19(9):1027–39. 29847195 10.1080/14656566.2018.1479742

[37] Mohan R, Chintala SK, Jung JC, Villar WV, McCabe F, Russo LA, Lee Y, McCarthy BE, Wollenberg KR, Jester JV, et al. Matrix metalloproteinase gelatinase B (MMP-9) coordinates and effects epithelial regeneration. J Biol Chem. 2002;277(3):2065–72. 11689563 10.1074/jbc.M107611200

[38] Lee D, Lee GW, Yoon SH. Relationship between ocular surface temperature and 0.1% cyclosporine a in dry eye syndrome with meibomian gland dysfunction. PLoS ONE. 2023;18(11):e0293472. 37983211 10.1371/journal.pone.0293472 PMC10659158

[39] Kim HY, Lee JE, Oh HN, Song JW, Han SY, Lee JS. Clinical efficacy of combined topical 0.05% cyclosporine A and 0.1% sodium hyaluronate in the dry eyes with meibomian gland dysfunction. Int J Ophthalmol. 2018;11(4):593–600. 29675376 10.18240/ijo.2018.04.09 PMC5902362

